# Variant profile of Brazilian patients with Sanfilippo syndrome type B

**DOI:** 10.1590/1678-4685-GMB-2025-0174

**Published:** 2026-06-26

**Authors:** Alice Brinckmann Oliveira Netto, Ana Carolina Brusius-Facchin, Kristiane Michelin‐Tirelli, Fernanda Medeiros Sebastião, Franciele Barbosa Trapp, Roberto Giugliani, Guilherme Baldo

**Affiliations:** 1 Universidade Federal do Rio Grande do Sul, Programa de Pós-Graduação em Genética e Biologia Molecular, Porto Alegre, RS, Brazil.; 2 Instituto de Genética para Todos, Porto Alegre, RS, Brazil.; 3 Hospital de Clínicas de Porto Alegre, Serviço de Genética Médica, Porto Alegre, RS, Brazil.; 4 Hospital de Clínicas de Porto Alegre, Serviço de Genética Médica, Rede MPS Brasil, Porto Alegre, RS, Brazil.; 5 DASA Genômica, São Paulo, SP, Brazil.; 6 Instituto Nacional de Genética Médica Populacional, Porto Alegre, RS, Brazil.; 7 Universidade Federal do Rio Grande do Sul, Departamento de Fisiologia, Porto Alegre, RS, Brazil.

**Keywords:** Mucopolysaccharidosis type IIIB, Brazil, novel variants

## Abstract

Mucopolysaccharidosis type IIIB (MPS IIIB, or Sanfilippo syndrome type B) is a lysosomal storage disorder caused by variants in the *NAGLU* gene, leading to heparan sulfate accumulation. This study analyzed 27 MPS IIIB Brazilian patients diagnosed via the MPS Brazil Network (2014-2022). Diagnosis involved biochemical tests [NAGLU enzyme activity, urinary glycosaminoglycans (GAG)], showing expected low activity of the enzyme and high concentration of GAGs. Molecular analysis of the *NAGLU* gene by Sanger sequencing or Targeted Next-Generation Sequencing confirmed the diagnosis. Forty-nine variants were found across patient alleles, comprising twenty-two different variants. Two variants were described for the first time: p.Gly79Arg and p.Leu598Pro (both missense). *In silico* tools predicted the novel variants as damaging/deleterious. The study identified 90.7% of the expected mutant alleles, observing variant heterogeneity and a higher frequency of missense variants. This characterization enhances understanding of the Brazilian MPS IIIB genetic landscape and is instrumental to the design of diagnostic and screening strategies.

Mucopolysaccharidosis type IIIB (MPS IIIB), or Sanfilippo syndrome type B (OMIM #252920) is a lysosomal storage disorder (LSD). It is one of the four subtypes of mucopolysaccharidosis type III (or Sanfilippo syndrome), being caused by variants in the *NAGLU* gene that encodes the N-acetyl-alpha-glucosaminidase (NAGLU) enzyme. The primary biochemical consequence of deficient NAGLU activity is the impaired degradation of a specific glycosaminoglycan (GAG), heparan sulfate, leading to its progressive accumulation within the lysosomes of various tissues and cells, most notably in the central nervous system (Zhao et al., 1996; Neufeld and Muenzer, 2019). The disease displays considerable clinical heterogeneity and variability in its presentation and progression (Zhao *et al.*, 1998; Weber et al., 1999; Coll et al., 2001). The condition is typically severe and progressive (Neufeld and Muenzer, 2019). Despite efforts to develop enzyme replacement therapy and gene therapy for MPS III, no specific treatment is available so far (Poswar et al., 2017, 2019; Wiesinger et al., 2025).

Accurate diagnosis of MPS IIIB is typically initiated through biochemical testing to measure the activity of the NAGLU enzyme (Marsh and Fensom, 1985), commonly performed on biological samples (cells or fluids). Also, urinary GAGs are important to analyze, especially to document its increase and identify the predominance of heparan sulfate. To further confirm the diagnosis, it is important to perform, whenever possible, the molecular analysis of the *NAGLU* gene (Yogalingam and Hopwood, 2001), which also allows carrier detection and prenatal diagnosis. The *NAGLU* gene is located on the long arm of chromosome 17 (17q21), comprising 6 exons and 5 introns, spanning approximately 8.3 kb. According to the Human Gene Mutation Database (HGMD), 292 different variants in the *NAGLU* gene have already been described, most of which being missense variants (73%) or small deletions (12%) (Stenson et al., 2003). These genetic alterations lead to reduced or complete loss of functional NAGLU enzyme activity, resulting in the accumulation of heparan sulfate (Neufeld and Muenzer, 2019). Techniques such as polymerase chain reaction (PCR), Sanger sequencing, next-generation sequencing (NGS) are utilized for the comprehensive detection and characterization of NAGLU gene variants (Brusius-Facchin et al., 2019; Montenegro et al., 2022).

Molecular genetic studies of MPS IIIB patients from diverse populations have demonstrated significant allelic heterogeneity (Beesley et al., 1998; Schmidtchen et al., 1998; Bunge et al., 1999; Coll et al., 2001; Emre et al., 2002; Pollard et al., 2013; Lin et al., 2018; Kong et al., 2020; Ozkinay et al., 2021) and have been instrumental in exploring genotype-phenotype correlations (Zhao et al., 1998; Weber et al., 1999). On the other hand, some studies have identified common *NAGLU* variants within specific populations, providing insights into their historical origins and prevalence. For instance, the c.700C>T p.(Arg234Cys) pathogenic variant is common in Portugal, accounting for 32% of Portuguese mutant alleles, and evidence suggests a single origin in the Iberian Peninsula (Mangas et al., 2008). Other common disease-causing variants includes c.419A>G p.(Tyr140Cys), found in different European populations, and c.1241A>G p.(His414Arg), which is relatively frequent in Greek families (Bunge *et al.*, 1999; Beesley *et al.*, 2004). In due course, the identification of pathogenic variants may be instrumental for the design of screening strategies, including neonatal screening. Sanfilippo syndrome is included in Brazil’s expanded neonatal screening program, which is approved but yet to be implemented in most of the country. 

In Brazil, the MPS Brazil Network (Rede MPS Brasil) was initiated in 2004 as a research project under the direction of Roberto Giugliani (Giugliani et al., 2016; Trapp et al., 2025). This network functions as an integrated system, supporting medical services nationwide. The primary goals of this initiative are to enhance diagnostic accessibility for MPS within the community and disseminate information concerning the clinical management of these disorders. The network processes a notable volume of diagnostic requests, receiving samples for the investigation of all MPS subtypes in patients primarily from Brazil, with diagnostic requests originated from foreign countries. This organizational and operational structure addresses factors contributing to the delayed diagnosis of MPS in Brazil, such as limited awareness among healthcare professionals and challenges in accessing diagnostic services (Giugliani *et al.*, 2016). In the present study, 27 patients diagnosed with Mucopolysaccharidosis type IIIB were evaluated between 2014 and 2022 through biochemical and molecular tests. The patient samples were part of a project approved by the HCPA’s Institutional Review Board (IRB0000921), which is recognized by the Office for Human Research. The data of this project is gathered in the MPS Brazil Network database. Considering this, we disclose that patients 10, 12, and 15 in this study correspond, respectively, to patients 29, 2, and 24 from the work previously published by our research group (Montenegro et al., 2022). The other individuals evaluated do not correspond between the cohorts.

The patients were born in different regions of Brazil, as illustrated in Figure 1. Clinical information was variably available across the centers; therefore, a systematic comparison of clinical features was not feasible. Future prospective data collection within the MPS Brazil Network aims to harmonize clinical reporting and enable more comprehensive genotype-phenotype correlation analyses.

**Figure 1-:**
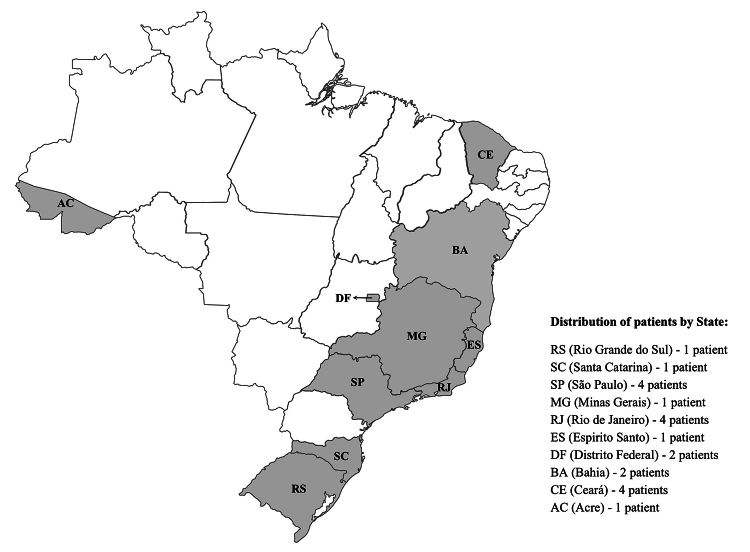
Map of Brazil illustrating the distribution of patients with MPS IIIB by state of birth.

Firstly, biochemical tests were performed through assessment of NAGLU enzyme activity in leukocytes, plasma or dried blood spots (DBS) using a fluorimetric assay, with a specific substrate as previously reported (Marsh and Fensom, 1985). Additionally, quantification of urinary GAGs, by the DMB method, and qualitative identification of GAGs species with mono-dimensional electrophoresis (de Jong et al., 1992) were performed.

To confirm the biochemical diagnosis, the samples were submitted to molecular analysis. The techniques used for such analyses were PCR followed by Sanger sequencing or Targeted Next-Generation Sequencing (TNGS). The purified PCR products were subjected to direct sequencing using ABI 3500xl 96 capillary DNA analyzer (Applied Biosystems^TM^) and the sequences were analyzed on BioEdit Sequence Alignment Editor. The TNGS was executed using a customized panel, that includes the NAGLU gene (Brusius-Facchin et al., 2019), in the Ion GenStudio^TM^ System with AmpliSeq^TM^ Library kit reagents (Thermo Fisher Scientific) (Oliveira Netto et al., 2021). 

To ensure the quality of the analyses, we use the following coverage parameters: mean coverage >100x and >98% of bases ≥20×. In the presence of regions with low coverage, the analysis of the region was confirmed by Sanger sequencing.

For the analysis of results, the Single Nucleotide Polymorphism database (dbSNP; http://www.ncbi.nlm.nih.gov/) and HGMD database (http://www.hgmd.org) were used to check the pathogenicity, as well as the description of the variants in the literature.

Given the extensive number of identified and novel NAGLU variants, *in silico* tools are widely used to predict their potential functional impact and aid in pathogenicity classification. Commonly utilized tools for this purpose include SIFT, which predicts whether an amino acid substitution will affect protein function based on sequence homology, PolyPhen/PolyPhen-2, which assesses the likelihood of a variant being probably damaging, and MutationTaster, which evaluates disease-causing potential (Mangas et al., 2008; Pollard et al., 2013; Hettiarachchi et al., 2018; Khorrami et al., 2019; Nasir Shalal et al., 2024). The predictions generated by these tools offer crucial evidence supporting the classification of variants, especially when integrated with other lines of evidence such as familial segregation data and the patient’s clinical presentation.

NAGLU activity information was available for all the patients and urinary GAGs levels for 21 of the 27 patients included in the study. GAGs electrophoresis was available for 23 patients and the most frequent excreted GAG identified was Heparan Sulfate. The enzyme activity values in MPS IIIB patients were lower than reference values and urinary GAGs were higher than reference values, as expected. In one patient, an atypical electrophoretic pattern (DS + HS) was observed, which could reflect secondary accumulation of dermatan sulfate or cross-reactivity in the qualitative assay (Table 1). Although MPS IIIB is classically associated with isolated HS accumulation, similar atypical qualitative GAG profiles have been reported in MPS subtypes, including MPS IIIB (Shi et al., 2014), particularly when semi-quantitative or colorimetric screening assays are used. Such findings have been attributed to secondary accumulation of other GAGs or to cross-reactivity inherent to qualitative electrophoretic methods. From a diagnostic perspective, atypical GAG patterns may complicate biochemical interpretation and reinforce the importance of confirmatory enzymatic and molecular testing in suspected MPS cases. No consistent genotype-phenotype correlation has been established linking the homozygous c.1336G>A variant identified in this patient to mixed GAG patterns, suggesting that this observation is more likely related to technical or metabolic variability rather than a specific genetic mechanism.

**Table 1-:** Biochemical and molecular information of the patients included in this work.

Patient	Allele 1	Allele 2	Enzyme¹	Quantification of GAGs [µg/mg creatinine]	GAGs Electrophoresis	Age at sample collection	Family data	State of birth
1	c.886delC p.(Leu296fs)	c.886delC p.(Leu296fs)	0.4 (P)	NP	HS	9	no information available	no information available
2	c.419A>G p.(Tyr140Cys)	-	0.21 (P)	28 mg/nmol (5.2-12)	no information available	8	no information available	no information available
3	c.503G>A p.(Trp168Ter)	c.700C>T p.(Arg234Cys)	0.2 (P)	NP	no information available	7	no information available	no information available
4	c.874G>A p.(Gly292Arg)	c.874G>A p.(Gly292Arg)	0.15 (P)	29 mg/nmol (5.7-13)	HS	8	no information available	no information available
5	c.1693C>T p.(Arg565Trp)	-	0.0 (P)	NP	HS	2	familial	ES
6	c.886delC p.(Leu296fs)	c.886delC p.(Leu296fs)	0.42 (P)	38 mg/nmol (5.7-13)	HS	9	no information available	no information available
7	c.607C>T p.(Arg203Ter)	-	0.15 (P)	600 (67-124)	HS	5	consanguinity	CE
8	c.1229T>C p.(Phe410Ser)	c.1420T>G p.(Trp474Gly)	0.26 (P)	NP	NP	26	no information available	SP
9	c.1597C>T p.(Arg533Ter)	c.1693C>T p.(Arg565Trp)	0.1 (P)	760 (44-106)	HS	7	no information available	RJ
10	c.1693C>T p.(Arg565Trp)	c.1876C>T p.(Arg626Ter)	0.8 (P)	NP	NP	6	no information available	RJ
11	c.419A>G p.(Tyr140Cys)	c.1927C>T p.(Arg643Cys)	0.30 (P)	164 (67-124)	HS	7	no information available	RS
12	c.1694G>A p.(Arg565Gln)	c.1694G>A p.(Arg565Gln)	0.0 (DBS)	230 (13-59)	HS	12	familial	CE
13	c.1694G>A p.(Arg565Gln)	c.1694G>A p.(Arg565Gln)	0.0 (DBS)	NP	HS	12	familial	CE
14	c.886delC p.(Leu296fs)	-	0.15 (L)	380 (79-256)	HS	2	no information available	no information available
15	c.1558C>T p.(Arg520Trp)	c.1558C>T p.(Arg520Trp)	0.2 (L)	1,954 (26-97)	no excretion	14	sporadic	MG
16	c.419A>G p.(Tyr140Cys)	c.2021G>A p.(Arg674His)	0.02 (L)	266 (53-115)	HS	6	sporadic	SP
17	c.700C>T p.(Arg234Cys)	c.700C>T p.(Arg234Cys)	0.0 (L)	505 (79-256)	HS	2	consanguinity	BA
18	c.607C>T p.(Arg203Ter)	c.607C>T p.(Arg203Ter)	0.5 (L)	176 (26-97)	HS	4	sporadic	CE
19	c.607C>T p.(Arg203Ter)	c.2186_2188delAAG p.(Lys728del)	0.21 (L)	251 (53-115)	HS	6	sporadic	AC
20	c.1597C>T p.(Arg533Ter)	c.1597C>T p.(Arg533Ter)	0.13 (L)	391 (67-124)	HS	4	consanguinity	RJ
21	c.886delC p.(Leu296fs)	c.886delC p.(Leu296fs)	0.05 (L)	655 (67-124)	HS	3	consanguinity	RJ
22	c.700C>T p.(Arg234Cys)	c.700C>T p.(Arg234Cys)	0.1 (P)	170 (26-97)	HS	9	consanguinity	BA
23	c.1793T>C p.(Leu598Pro)	c.235G>C p.(Gly79Arg)	0.5 (L)	439 (67-124)	HS	4	no information available	DF
24	c.192del p.(Tyr65fs)	-	0.5 (L)	361 (67-124)	HS	5	consanguinity	SP
25	c.1318G>C p.(Gly440Arg)	c.1834A>G p.(Ser612Gly)	0.19 (L)	77 (13-45)	HS	22	sporadic	SP
26	c.886delC p.(Leu296fs)	c.886delC p.(Leu296fs)	0.25 (P)	181 (44-106)	HS	8	sporadic	SC
27	c.1336G>A p.(Glu446Lys)	c.1336G>A p.(Glu446Lys)	2.6 (L)	164 (44-106)	DS + HS	8	consanguinity	DF

L: Leukocytes; P: Plasma; DBS: Dried Blood Spot; -: not found; NP: not performed; HS: Heparan Sulfate; DS: Dermatan Sulfate.

¹Enzyme reference values: DBS (0.96-5.80 nmol/h/ml); L (10-34 nmol/17h/mg protein); P (11-37 nmol/h/ml); (GAGs reference value).

Reference sequences: NM_000263.3 (transcript) / NP_000254.2 (protein)

Molecular analysis identified 22 different variants among the 27 patients diagnosed with MPS IIIB and 49 variants were detected in total. Two variants are described for the first time in this study, c.235G>C (p.Gly79Arg) and c.1793T>C p.(Leu598Pro), both being missenses (Table 2). *In silico* prediction tools were used to evaluate the pathogenicity of the novel variants (Table 3). Although the novel variants were identified in patients with a biochemical profile consistent with MPS IIIB and predicted as deleterious by multiple *in silico tools*, no segregation or functional validation was possible due to sample limitations. The two novel variants identified in this study were classified according to ACMG guidelines (Richards et al., 2015) as variant of uncertain significance (VUS), in agreement with the classifications presented in Table 3. Nonetheless, we acknowledge that segregation analysis or functional studies would further strengthen the pathogenicity assessment, particularly considering that both are currently classified as VUS. The high frequency of the p.(Leu296fs) variant (18% of the alleles identified in this cohort) caught our attention, raising the possibility of a founder effect within the Brazilian population. Future haplotype analysis would be valuable to determine whether these cases derive from a single ancestral mutation event or represent multiple independent occurrences. Excluding one patient, which did not have the second variant identified, all other patients bearing it are homozygous. In one of them, historical records indicate consanguinity. For most of the other cases, we do not have the consanguinity information available nor the origin of the patient. Therefore, although consanguinity may help explain our findings in part, we cannot reach a conclusion based on the limited data available.

**Table 2 -:** Summary of the variants and their corresponding frequency found in this study.

Location	Nucleotide change	Effect on protein	Alleles	Reference
**Missense**			29	
**exon 1**	**c.235G>C**	**p.Gly79Arg**	**1**	**this report**
exon 2	c.419A>G	p.Tyr140Cys	3	Zhao et al., 1998
exon 4	c.700C>T	p.Arg234Cys	5	Beesley et al., 1998
exon 5	c.874G>A	p.Gly292Arg	2	Bunge et al., 1999
exon 6	c.1229T>C	p.Phe410Ser	1	Weber et al., 1999
exon 6	c.1318G>C	p.Gly440Arg	1	Montenegro et al., 2021
exon 6	c.1336G>A	p.Glu446Lys	2	Beesley et al., 2004
exon 6	c.1420T>G	p.Trp474Gly	1	Coll et al., 2001
exon 6	c.1558C>T	p.Arg520Trp	2	Tessitore et al., 2000
exon 6	c.1693C>T	p.Arg565Trp	3	Weber et al., 1999
exon 6	c.1694G>A	p.Arg565Gln	4	Bunge et al., 1999
**exon 6**	**c.1793T>C**	**p.Leu598Pro**	**1**	**this report**
exon 6	c.1834A>G	p.Ser612Gly	1	Zhao et al., 1998
exon 6	c.1927C>T	p.Arg643Cys	1	Weber et al., 1999
exon 6	c.2021G>A	p.Arg674His	1	Zhao et al., 1996
**Nonsense**			9	
exon 2	c.503G>A	p.Trp168Ter	1	Coll et al., 2001
exon 3	c.607C>T	p.Arg203Ter	4	Schmidtchen et al., 1998
exon 6	c.1597C>T	p.Arg533Ter	3	Mangas et al., 2008
exon 6	c.1876C>T	p.Arg626Ter	1	Zhao et al., 1996
**Small deletions and duplications**			11	
exon 1	c.192del	p.Tyr65fs	1	Beesley et al., 1998
exon 6	c.886delC	p.Leu296fs	9	Montenegro et al., 2022
exon 6	c.2186_2188delAAG	p.Lys728del	1	Pollard et al., 2013
Total			49	

Reference sequences: NM_000263.3 (transcript) / NP_000254.2 (protein).

**Table 3 -:** Bioinformatic analysis of the novel variants identified in this work.

Coding sequence	Protein sequence	ACMG Classification	ACMG criteria	gnomAD frequency^1^	SIFT^2^	PolyPhen^3^	Mutation Taster^4^
c.235G>C	p.(Gly79Arg)	VUS	PM1, PP3	not reported	damaging	0.992	deleterious
c.1793T>C	p.(Leu598Pro)	VUS	PM2, PP3	0.0000006217	damaging	0.990	deleterious

Reference sequences: NM_000263.3 (transcript) / NP_000254.2 (protein). ^1^https://gnomad.broadinstitute.org/; ^2^http://genetics.bwh.harvard.edu/pph2/; ^3^https://sift.bii.a-star.edu.sg/;

^4^ https://www.genecascade.org/MutationTaster2021/.

MPS IIIB is characterized by prominent genetic heterogeneity. This variant heterogeneity is intricately linked to the wide variation in the clinical phenotype observed in patients with MPS IIIB (Yogalingam and Hopwood, 2001; Emre et al., 2002; Andrade et al., 2015). Phenotypes can range from severe forms, often associated with nonsense, insertion, or deletion variants that result in truncated proteins, to more attenuated phenotypes that may retain some residual enzymatic activity (Yogalingam and Hopwood, 2001).

The *NAGLU* gene from the cohort of 27 Brazilian Sanfilippo B patients was analyzed and 90.7% of the expected disease-causing alleles were identified. In four patients only one variant was found in heterozygosis, therefore the second one could be in a region that is not covered by the techniques used in this study. These may include deep intronic variants affecting splicing, large deletions or duplications not detectable by standard sequencing approaches, or variants in promoter or regulatory regions. The incorporation of complementary methodologies, such as MLPA or RNA-based analyses, may improve detection rates in future studies. According to the literature, we observed variant heterogeneity among our sample (Weber et al., 1999) and a higher frequency of missense variants compared to other variant types.

Studies involving targeted sequencing frequently leverage these bioinformatics approaches to evaluate novel or previously uncharacterized variants. The partial validation of newly diagnosed pathogenic variants through *in silico* analyses contributes significantly to the expansion and updating of genetic databases concerning Sanfilippo disease variations and NGS gene panels, ultimately improving the efficiency and accuracy of genetic counseling for rapid risk examinations and disease surveillance (Khorrami et al., 2019). Knowledge about mutational profile may improve the performance of screening protocols, including neonatal screening in asymptomatic babies, to be implemented when transforming therapies become available. While NAGLU activity was below reference values in all cases, molecular analysis using this approach did not allow the identification of both alleles in 5 of 27 patients. Considering this data and given the heterogeneity of variants found, a neonatal screening based on biochemical analysis seems more appropriate for the Brazilian population. 

Even though Sanfilippo syndrome is included in Brazil’s expanded newborn screening program, nationwide implementation remains heterogeneous, with substantial regional variability in access and coverage (de Souza et al., 2025). The marked allelic heterogeneity observed in this study supports the prioritization of biochemical screening as a first-tier approach for MPS IIIB in Brazil. Nonetheless, the identification of p.(Leu296fs) as the most frequent variant may inform future tiered screening strategies combining biochemical assays with targeted molecular testing. While the 90.7% molecular detection rate observed in this cohort is encouraging for diagnostic confirmation, it remains below the threshold typically required for standalone molecular newborn screening, further supporting a combined biochemical-molecular approach.

## Data Availability

The dataset that supports the results of this study is not publicly available.
